# 

**Published:** 1900-06-09

**Authors:** 


### NOTICE TO CORRESPONDENTS.

All MS., Letters, Books for Review, and other matters intended for the Editor should be addressed The Editor, "The Hospital," The Hospital Building, 28 & 29, Southampton Street, Strand, W.C.

The Editor cannot undertake to return rejected MS., even when accompanied by stamped directed envelope.

**Notice to Advertisers and Subscribers.**

All Advertisements, Orders for Copies of The Hospital, and Business Communications should be addressed to **THE MANAGER (Not to the Editor)**, The Hospital, The Hospital Building, 28 & 29, Southampton Street, Strand, London, W.C.

The Cost of Subscription, post free, is as follows:—Per week, 2½d.; per quarter, 2s. 8d.; per half-year, 5s. 3d.; per annum, 10s. 6d. Foreign:—Per annum, 15s. 2d., payable, in all cases, in advance.

**NOTICE.—Vol. XXVII. of "The Hospital" (October, 1899, to April, 1900), in handsome cloth binding, is now on sale at the Office of this Journal, price 7s. 6d. Cases for binding the Numbers contained in this Volume 1s. 6d. Index to Vol. XXVII., 2½d., post free.**

**The Monthly Part for June is now ready. Price 6d., by post 8d.**

### BOOKS RECEIVED.

Bemrose and Sons.
"Lateral Curvature of the Spine." By Geo. Steele Perkins, M.D.

William Blackwood and Sons.

"Mona Maclean, Medical Student." By Graham Travers (Margaret Todd, M.D.)

Sampson Low, Marston, and Co.
"Twentieth Century Practice of Modern Medical Science." Edited by Thomas L. Stedman, M.D. Volume XIX.

Henry Glaisher.
"Practical Gynæcology: A Handbook of the Diseases of Women." By Heywood Smith, M.A., M.D., Oxon. Second edition.

Sealey, Bryers, and Wather.
"Lunacy: The Rising Tide." By W. J. Corbet, M.P.

G. W. Bacon and Co.
"John Bull and his Friends." By Fred. W. Rose.

Her Majesty's Stationery Office.
"Report of the Medical Officer of the Local Government Board."

Baillière, Tindall, and Cox.

"Treatment by Hypnotism and Suggestion; or, Psycho-Therapeutics." By C. Lloyd Tuckey, M.D. Fourth edition.

Percival Jones, Birmingham.

"Report on the Health of the City of Birmingham for 1899." By Alfred Hill, M.D., &c., Medical Officer of Health.

Robertson and Gray, Coventry.

"Annual Report on the Health of the City of Coventry for 1899." By Hugh Snell, M.D., &c., Medical Officer of Health.

### THE NURSE'S REPORT BOOK.

Foolscap 4to., Black cover, **6d.** net. By Post 8d.

**Compiled by K. H. (Cert.)**

CONTAINING space for general details at the beginning of the book, and for general remarks on the case at the end of the book, and with space for 200 entries of each of the following details: Time—Nourishment—Stimulants—Temperature—Pulse—Respiration—Urine—B.O.—Sleep—Remarks.

H. J. GLAISHER, 57, Wigmore Street, Cavendish Square, W.

### BOOKS ON NURSING.

**A COMPLETE SYSTEM OF NURSING.** Written by various contributors, consisting entirely of Medical Men and Nurses. Edited by HONNOR MORTEN, Author of "The Nurse's Dictionary," &c. 8vo., cl., 7s. 6d. *net.*
"We are bound to say that the greater part of this handy volume contains much that is useful and interesting. . . . Lastly, the list of recipes for invalid cookery is both original and serviceable."—*The Nurses' Journal.*
"As a book of reference, a nurse may find in its pages exactly the information she may want to resolve a doubt or difficulty."—*Glasgow Herald.*
"The chapter on 'The Nursing of Babies' is admirable, and should be read by all mothers as well as nurses."—*The Hospital.*

**WOUNDED IN WAR.** Surgeon's Handbook of Treatment. By F. ESMARCH, translated by H. H. CHILTON. With coloured plates and 536 engravings. 8vo., £1 8s.

**MANUAL OF FIRST AID.** Being a Text-book for Ambulance Classes and a Work of Reference for Domestic and General Use. By Dr. J. A. AUSTIN, Lecturer to St. John's Ambulance Association, and Author of "Ambulance Sermons." Illustrated with diagrams. Dedicated by permission to H.R.H. Princess Christian of Schleswig-Holstein. Crown 8vo., 3s. 6d. [*Ready.*
"Dr. Austin's little book is well and interestingly written. . . . The plan of the book follows the syllabus of the St. John's Ambulance Association, and is copiously illustrated with diagrammatic figures, which are plain and simple and accurate. Altogether the book is a good specimen of its class."—*Lancet.*

**HOW TO TREAT ACCIDENTS AND ILLNESSES.** A Handbook for the Home. By HONNOR MORTEN, Author of "Sketches of Hospital Life," &c. Second Edition. With illustrations. Post 8vo., boards, 2s.; cloth, 2s. 6d.
"A very sensible 'handbook for the home,' prepared by a practical person, presumably a lady with nursing experience. It tells what to do in case of common accidents, common emergencies, and the various common illnesses to which flesh is heir. The explanations are given in the simplest business-like language, and the incidental advice is full of common-sense, and tends to reassurance of the patient. To the systematic treatment of the various physical troubles liable to afflict a family are appended some useful general hints on nursing and on the preservation of health."—*Daily Chronicle.* "Just such a book as a nurse or a good housewife needs."—*Scotsman.*

**LYING-IN.** A Book for Every Mother. By MARIAN HUMFREY, of the British Lying-In Hospital, London, and Author of "A Manual of Obstetric Nursing." Small crown 8vo., paper covers, 1s.
DR. ALFRED HILL (Medical Officer of Health, Birmingham) says:—"I have gone through the book, and am convinced that if lying-in women will follow its directions, the discomforts, dangers, and diseases which too often attend the natural process will be either avoided or mitigated."

**1. A MANUAL OF OBSTETRIC NURSING.** By MARIAN HUMFREY. Part I.—MATERNAL. Part II.—INFANTILE. Crown 8vo., cloth, 3s. 6d. Dedicated by permission to H.R.H. the late Princess Mary of Cambridge, Duchess of Teck.
*The Hospital* says: "This volume is certainly a very valuable addition to the literature of obstetric nursing. Not only is every little detail mentioned, but its special excellence consists in the very lucid manner in which all is explained. . . . The arrangement of the chapters, and the advice given are beyond praise."

**2. A MANUAL OF OBSTETRIC NURSING.** By MARIAN HUMFREY. Vol. II. Part I.—MATERNAL. Part II.—INFANTILE. Crown 8vo., cloth, 3s. 6d. This volume treats of duties, special to the gravest emergencies, of Obstetric Nursing, and thus completes the curriculum of instruction.

LONDON: SAMPSON LOW, MARSTON & COMPANY, LIMITED., St. Dunstan's House, Fetter Lane, E.C.

